# Correction to “Bioimpedance Measurement for Monitoring Chronic Wounds: A Systematic Review”

**DOI:** 10.1111/iwj.70751

**Published:** 2025-08-19

M. Antoszewska , K. Połomska , P. Spychalski , A. Kekonen , J. Viik , and W. Barańska‐Rybak , “Bioimpedance Measurement for Monitoring Chronic Wounds: A Systematic Review,” International Wound Journal 22 (2025): e70707, 10.1111/iwj.70707.40505690 PMC12162137

In the original publication, the following sentence was inadvertently added during revision, which was generated via a GenAI tool: “Let me know if you would like variations in tone (e.g., more technical, formal or simplified).”

The following Acknowledgements statement is now included for the article:

